# The percentage of lepidic growth is an independent prognostic factor in invasive adenocarcinoma of the lung

**DOI:** 10.1186/s13000-015-0335-8

**Published:** 2015-07-09

**Authors:** Trond-Eirik Strand, Hans Rostad, Erik H. Strøm, Philip Hasleton

**Affiliations:** Department of Registration, Cancer Registry of Norway, Oslo, Norway; Department of Pathology, Oslo University Hospital, Oslo, Norway; Department of Inflammation and Repair, University of Manchester, Manchester, United Kingdom; Department of Pathology, Hebrew University Medical School, Jerusalem, Israel

**Keywords:** Adenocarcinoma in situ (AIS), Lepidic growth, Prognosis, Population-based survival, Lepidic predominant adenocarcinoma (LPA) Bronchioloalveolar carcinoma (BAC)

## Abstract

**Background:**

The literature is inconclusive as to whether the percentage of the lepidic component of an invasive adenocarcinoma (AC) of the lung influences prognosis. We studied a population-based series of selected, resected invasive pulmonary ACs to determine if incremental increases in the lepidic component were an independent, prognostic variable.

**Methods:**

Patients undergoing resection for lung cancer reported to the Cancer Registry of Norway and diagnosed in the period 1993-2002 with a bronchioloalveolar carcinoma (BAC) (old terminology) (adenocarcinoma in situ, AIS in the new terminology) in the lung were selected. A pulmonary pathologist reviewed all sections and estimated the percentage of the lepidic component. Follow-up of survival was to the end of 2013.

**Results:**

One hundred thirty-one patients were identified, 102 had AC with lepidic growth. Of these, 44 had AC with a component of lepidic growth less than 50 % and seven had AC with 95 % lepidic component or more. One of the latter cases was considered to be AIS. In regression analyses, superior survival was associated with a greater lepidic component (p = 0.041). Mucinous tumors had a worse prognosis than non-mucinous (p = 0.012) in regression analyses, as did increasing age and stage. The five-year observed survival was 69.0 % for non-mucinous cases and 66.7 % for the group with a lepidic component of 80 % or greater.

**Conclusion:**

The percentage of the lepidic component appears to be an independent, significant prognostic factor in a selection of pulmonary AC.

## Background

New concepts were introduced in the 2011 classification of lung adenocarcinomas (ACs) by the International Association for the Study of Lung Cancer, American Thoracic Society, and European Respiratory Society [1]. One was the lepidic-predominant adenocarcinoma (LPA) for diagnoses based on resections. This property was first noted by Noguchi in 1995 [2] and has later been found to indicate a favorable prognosis [3] and with 5-year disease free survival of 94 % [4].

When LPA was introduced in the 2011 classification, histologic subtyping to assess the different patterns semi-quantitatively in 5 % increments and describing a single predominant pattern for the diagnosis were suggested. The recording of the percentage of a tumor component was classified in the paper as ‘a weak recommendation’ with ‘low-quality evidence,’ probably because of the lack of research on this topic.

The association between the percentage of lepidic component and prognosis is not well known. Recently Kadota and colleagues reported that higher percentage of lepidic pattern correlated with lower risk of recurrence [5]. Takahashi and colleagues studied AC patterns (including LPA) quantitatively but the effect of the increments in the lepidic component was not specified other than for the groups predominant and not-predominant [3]. Also, quantification of the component has been used to estimate the non-lepidic tumor diameter, which proved useful prognostically [6, 7].

The aim of the current paper is to investigate the effect of the percentage of the lepidic component as an independent prognostic factor in a population-based series of pulmonary resections reported to the cancer registry as ‘bronchioloalveolar carcinoma’ (‘BAC’). This entity is now termed adenocarcinoma in situ (AIS), if there is no invasion.

## Methods

19,688 patients reported to the Norwegian Cancer Registry were diagnosed with lung cancer in Norway in the period 1993-2002. Among these, 3,306 lung resections were performed on 3,286 patients. A total of 5,113 cases was diagnosed as adenocarcinoma (26 %) in this period and of these 1,396 were resected. A diagnosis of LPA or BAC (this diagnosis made before the new nomenclature proposal in 2011 [1]) was reported to the Cancer Registry in 267 cases (0.5 % of cases diagnosed as adenocarcinoma). 131 of these 267 cases were resected and thus further investigated.

Of these 131 cases initially reported as LPA or BAC, all histological sections were reviewed by one pulmonary histopathologist (PH), blinded from any clinical data. The new international multidisciplinary classification of pulmonary AC was used [1]. Only haematoxylin and eosin (H & E) sections were available, unless special stains were provided. All the sections, including normal lung, were reviewed; the major type of AC present was noted, as well as other subtypes. The percentage of the lepidic component was estimated semi-quantitatively in five percentage point increments on all sections from any case, rounded up and the mean determined for the total number of slides for the case. The cell type (mucinous and/or non-mucinous) of the lepidic component was noted. Sex, age, location of the tumor (left or right side), tumor size, as measured macroscopically, and pTNM (7^th^ ed. data) were available for all cases but these details were not known by the reviewing pathologist.

Cases with a primary lung cancer other than AC, AC with no lepidic component or where it was impossible to determine the histological subtype were excluded. Cases with AIS were excluded from survival analyses. AIS cases were classified with T-descriptor according to tumor size and other features and not limited to Tis.

Univariate analyses were performed with the Chi-square test. The Kruskal Wallis test was used to compare non-parametric tumor sizes between the different groups. Observed survival was estimated using the life table method and calculated from the date of surgery to death, emigration from the country or end of follow-up (December 31 2013).

Multivariate Cox regression analysis was used to identify important survival factors. Included in the Cox analysis were age (continuous), sex, side of resection (right/left), surgical procedure and technique (sublobar resection/lobectomy/bilobectomy and pneumonectomy), tumor pStage (I-IV), tumor size (continuous)(whole tumor as measured by the initial pathologist), proportion of the lepidic component in percentage (continuous) and cell type in the lepidic component (mucinous/non-mucinous/combination). Pleural invasion is known to be an adverse prognostic factor [8–11]. However, it was not analysed as an independent variable since it would introduce an interaction with stage.

The software package R version 3.2.0 was used for statistical analyses. (Foundation for Statistical Computing, Vienna, Austria; http://www.R-project.org).

Virtually all Norwegian cancers are reported to the Cancer Registry [12]. Norwegian legislation does not require informed patient consent for studies based on data from central registries. The study was approved by the regional ethics committee.

## Results

**Fig. 1:**
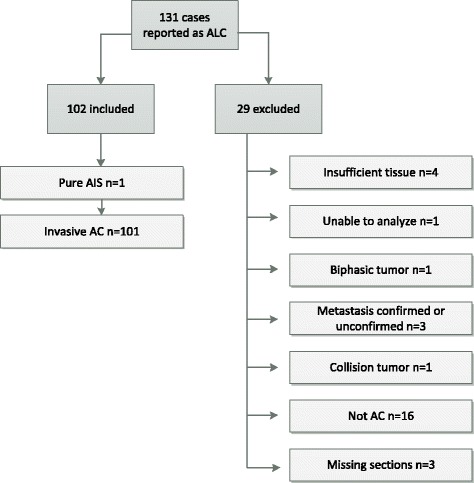
Overview of inclusion and exclusion of patients

Of the 131 cases initially reported as ‘BAC’; twenty nine were excluded (Fig. 1). The remaining 102 cases were classified as adenocarcinomas with a lepidic component (ALC) not BAC.

The mean age for patients with ALC was 65.2 years (men 64.9, women 65.5). The number of sections per case, including non-tumor, ranged from 2 to 35 (average, 5.3 sections/case).

**Fig. 2:**
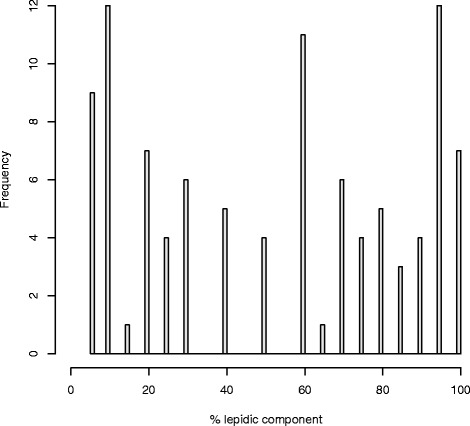
Proportion of lepidic component in resected patients with lepidic predominant adenocarcinoma

The distribution of the lepidic component ranged from 5-100 % and the histogram is displayed in Fig. 2. Of the ALCs, one was regarded adenocarcinoma in situ (AIS) with a pure lepidic component, the tumor size was 1.8 centimeter in diameter and it was pStage IA. Another six patients had lepidic components ranging from 96-100 %. No minimal invasive adenocarcinoma (MIA) was identified.

The remaining 96 cases had invasive AC with a varying lepidic component up to and including 95 %. In one case, it was impossible to determine the percentage of lepidic growth, in a predominantly papillary AC.

The characteristics (age, sex, side involved, pStage and type of the lepidic component) for cases with ALC according to the percentage of the lepidic component are presented in Table 1. In 13 cases the cell type in the lepidic component had both mucinous and non-mucinous cells lining the alveolar walls.

**Table 1:** Characteristics of adenocarcinoma with a lepidic component by percentage of the lepidic component

	Lepidic component				
	<50 %	50-75 %	80-95 %	96-100 %	p-value
Total	44 (44)	26 (26)	24 (24)	7 (7)	
Sex					0.49
Female	21 (39)	13 (24)	15 (28)	5 (9)	
Male	23 (49)	13 (28)	9 (19)	2 (4)	
Age (years)					0.43
<50	2 (33)	1 (17)	1 (17)	2 (33)	
50-59	10 (44)	7 (30)	4 (17)	2 (9)	
60-69	16 (47)	7 (21)	9 (27)	2 (6)	
70+	16 (42)	11 (29)	10 (26)	1 (3)	
Side					0.11
Left	14 (36)	8 (21)	12 (31)	5 (13)	
Right	30 (48)	18 (29)	12 (19)	2 (3)	
pStage					0.71
I	29 (45)	14 (22)	16 (25)	6 (9)	
II	11 (46)	7 (29)	5 (21)	1 (4)	
III	4 (33)	5 (42)	3 (25)	0 (0)	
Lepidic type					0.0024
Mucinous	5 (29)	2 (12)	5 (29)	5 (29)	
Non-mucinous	35 (49)	20 (28)	14 (20)	2 (3)	
Mucinous and non-mucinous	4 (31)	4 (31)	5 (39)	0 (0)	
Tumor size (centimeter)					0.64
<3	23 (52)	9 (21)	10 (23)	2 (5)	
3-5	14 (33)	14 (33)	10 (24)	4 (10)	
5+	7 (47)	3 (20)	4 (27)	1 (7)	

The mean tumor size in non-mucinous cases with ALC (n = 71) was 3.1 cm, in mucinous cases (n = 17) 5.8 cm and in mixed cases (mucinous & non-mucinous, n = 14) 3.9 cm. The sizes were significantly different between these groups (p = 0.010).

**Fig. 3:**
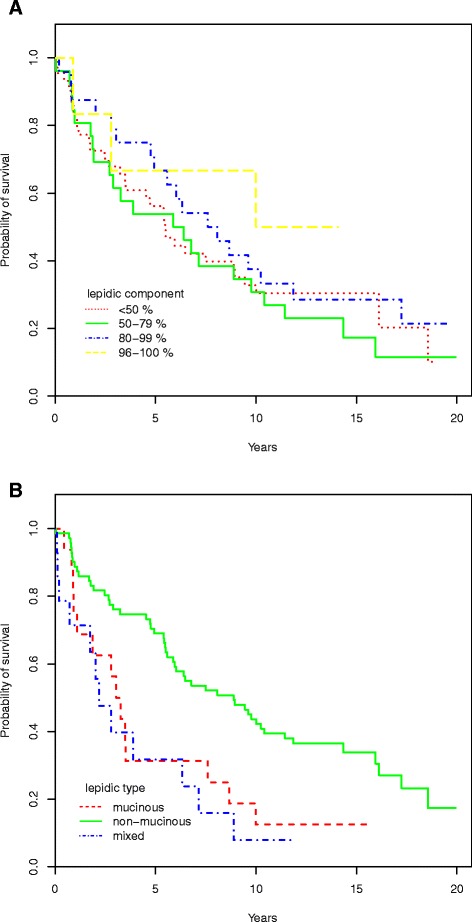
Survival according to proportion of lepidic component (**a**) and type (**b**)

The 1-, 2-, 5- and 10-year survivals, as well as the influence of the varying lepidic component percentages and type are shown in Table 2 and Fig. 3. The two groups with highest percentage of lepidic component had a moderately improved survival compared to the two groups with the lowest percentage. In univariate analyses the mucinous subtype appeared to have a worse prognosis, although the confidence intervals had a small overlap.

**Table 2:** Survival according to the lepidic component and type

	N	1 yr. (95 % CI)	2 yr. (95 % CI)	5 yr. (95 % CI)	10 yr. (95 % CI)
Total	101	84.2 (77.3-91.6)	76.2 (68.3-85.0)	58.1 (49.2-68.6)	34.1 (25.9-44.8)
Lepidic component					
<50 %	44	84.1 (73.9-95.6)	72.6 (60.5-87.1)	56.2 (43.2-73.1)	32.8 (21.4-50.3)
50-79 %	26	80.8 (67.0-97.4)	72.6 (60.5-87.1)	56.2 (43.2-73.1)	32.8 (21.4-50.3)
80-95 %	24	87.5 (75.2-100)	87.5 (75.2-100)	66.7 (50.2-88.5)	37.5 (22.4-62.9)
96-100 %	6	83.3 (58.3-100)	83.3 (58.3-100)	66.7 (37.9-100)	50.0 (22.5-100)
Unknown	1				
Lepidic type					
Mucinous	16	75.0 (56.5-99.5)	62.5 (42.8-91.4)	31.2 (15.1-64.6)	12.5 (3.4-45.7)
Non-mucinous	71	88.7 (81.7-96.4)	81.7 (73.2-91.2)	69.0 (59.1-80.7)	43.7 (33.5-56.9)
Mucinous and non-mucinous	14	71.4 (51.3-99.5)	63.5 (42.4-95.1)	31.8 (14.2-70.8)	7.9 (1.2-51.9)

Yr. - Year, CI - Confidence interval

Multivariate analyses demonstrated that the independent variables important for survival were age, pStage, the percentage and type of the lepidic component (Table 3). No interactions were found, and proportional hazards were not violated.

**Table 3:** Cox regression

	N	HR	p-value
Age (continuous)	100	1.04	0.0094
Sex			0.76
Female	53	1.00	
Male	47	0.92	
pStage			0.0014
I	64	1.00	
II	24	2.63	
III	12	4.81	
Side			0.58
Left	39	1.00	
Right	61	1.18	
Procedure			0.66
Sublobar resection	3	1.00	
Lobectomy	78	0.81	
Pneumonectomy or bilobectomy	19	1.10	
Tumor size (continuous)	100	0.99	0.88
Lepidic component % (continuous)	100	0.99	0.041
Lepidic cell type			0.012
Mucinous	16	1.00	
Non-mucinous	71	0.36	
Mucinous and non-mucinous	13	0.99	

HR – Hazard ratio

## Discussion

This study demonstrates an independent, positive prognostic association with incremental increases in the percentage of the lepidic component associated with selected cases of invasive pulmonary AC. The five-year observed survival rate was 56 % in the two groups classified with lepidic component percentage less than 80 % and 67 % in the two groups with 80 % or more despite the latter groups having a higher proportion of mucinous cases.

Increasing proportions of the mucinous lepidic type among the group with the highest percentage of lepidic growth as well as not using cancer-specific death as endpoint for survival could explain why the 5-year survival for these groups was not close to 100 %, as reported by others [13]. Many cases had both a mucinous and non-mucinous lepidic component, suggesting the neoplastic process was differentiating along two cell pathways. Divergent growth patterns are common in lung carcinogenesis [14].

Five studies have previously quantitated the percentage of the BAC component but not in the same way as this investigation [3, 15–18]. Carretta et al. in patients with Stage IA disease with > 50 % ‘BAC’ component had an 83 % 5-year survival compared to 29 %, with IB disease and a ‘BAC’ component ≤ 50 % [18]. Higashiyama et al. showed patients with < 50 % ‘BAC’ component in their study had a poorer prognosis than those with > 50 % [15]. Yokose et al. demonstrated tumors of 3 cm. or less with > 75 % lepidic growth and a central focus of fibrosis 5 millimeters or less in maximum diameter had a 100 % seven year survival [16]. These early invasive tumors are smaller than this series. Okubo et al. conversely found a worse prognosis in cases with 100 % ‘BAC’, than tumors with a mixture of ‘BAC’ and AC [17]. This study included a range of operations from wedge resections to pneumonectomies. Twenty-two per cent of their cases were mucinous, which would have influenced prognosis. One-hundred and thirteen of 119 of these cases had aerogenous growth. A pure ‘BAC’ pattern was seen in only 17 patients. All the studies pre-date the latest guidelines and it is probable that ‘BAC’ has been overdiagnosed pathologically

Ebbert and colleagues did not quantify the percentage of the BAC component and reported 1- and 5-year survival for 78 pure ‘BAC’ patients were 95 % and 84 % respectively, and for the 178 “non-pure” patients, 93 % and 46 %, respectively [19]. Restaging for pure ‘BAC’ cases resulted in 9/78 cases (12 %) changing stage. Compared to the old staging, patients with advanced stage under the new staging procedure had a worse 5-year survival, (53 % vs. 45 %) but no change was observed for stage IA. Of the 338 patients, initially diagnosed as ‘BAC’, 117 were reclassified by an experienced pulmonary pathologist as “pure” and 221 were ‘non-pure’ BAC [20]. As in our series, tumors diagnosed as BAC on the basis of cytology or needle biopsy specimens were excluded, but still as many as 44 patients (43 %) had less than 50 % lepidic component and several of these would not meet the new classification criteria for AIS. Many Western cases reported from large centers as ‘BAC’ has formerly been re-diagnosed as invasive AC [21].

The reproducibility in pathologist’s diagnosis of the different histologic patterns of pulmonary AC should also be considered. In a recent study the reproducibility was high (k = 0.77) when 26 pathologists studied the primary patterns of this tumor [21]. Thus while this study should be repeated for verification, a strength with the current study is that only one histopathologist assessed every case.

Comparisons with older series are difficult since they were often diagnosed using old terminology, as mixed series of ‘BAC; and AC, in addition, mucinous and non-mucinous subtypes were not analyzed separately.

Although this series is population-based and represented all resected cases in the country reported with a diagnosis of ‘BAC’, there is a possible selection bias inherent in the study in that resected cases with a lepedic component without being classified as BAC would not be eligible for the inclusion criteria. We do not know the magnitude but according to the histogram in Fig. 2 the distribution indicates the study population is representative for assessing degree of lepidic component.

Small tumors, 3 cm or less in diameter with a predominant lepidic component must be sampled in their entirety to exclude either AIS or AIS having foci of invasion (minimally invasive AC). This is another limitation of the present study, as the entire tumor and surrounding lung tissue do not appear to have been sampled.

## Conclusion

This study shows that in pulmonary AC the specific percentage of the lepidic component seems to be an independent, significant prognostic factor.
